# Modulation of Neuronal Proteome Profile in Response to Japanese Encephalitis Virus Infection

**DOI:** 10.1371/journal.pone.0090211

**Published:** 2014-03-05

**Authors:** Nabonita Sengupta, Sourish Ghosh, Suhas V. Vasaikar, James Gomes, Anirban Basu

**Affiliations:** 1 National Brain Research Centre, Manesar, Haryana, India; 2 Kusuma School of Biological Sciences, Indian Institute of Technology Delhi, New Delhi, India; National Institute of Allergy and Infectious Diseases - Rocky Mountain Laboratories, United States of America

## Abstract

In this study we have reported the *in vivo* proteomic changes during Japanese Encephalitis Virus (JEV) infection in combination with *in vitro* studies which will help in the comprehensive characterization of the modifications in the host metabolism in response to JEV infection. We performed a 2-DE based quantitative proteomic study of JEV-infected mouse brain as well as mouse neuroblastoma (Neuro2a) cells to analyze the host response to this lethal virus. 56 host proteins were found to be differentially expressed post JEV infection (defined as exhibiting ≥1.5-fold change in protein abundance upon JEV infection). Bioinformatics analyses were used to generate JEV-regulated host response networks which reported that the identified proteins were found to be associated with various cellular processes ranging from intracellular protein transport, cellular metabolism and ER stress associated unfolded protein response. JEV was found to invade the host protein folding machinery to sustain its survival and replication inside the host thereby generating a vigorous unfolded protein response, subsequently triggering a number of pathways responsible for the JEV associated pathologies. The results were also validated using a human cell line to correlate them to the human response to JEV. The present investigation is the first report on JEV-host interactome in *in vivo* model and will be of potential interest for future antiviral research in this field.

## Introduction

Viral entry, replication, and assembly are dynamic processes that involve numerous host-pathogen interactions. Traditional antiviral drug-discovery approaches were directed against virally encoded enzymes that are essential for viral replication but have failed to be effective. Viruses have the inherent capacity to rapidly mutate and become resistant to even the most potent of drugs because of their low-fidelity replication mode [1]. Hence, the focus of antiviral research is shifting towards manipulating the host antiviral response by targeting cellular enzymes or cofactors required for the viral life cycle.

Viruses are known to enter host cells via various receptor-mediated/endocytosis-mediated routes and replicate in the cytoplasm of infected cells. During the process, they invade various host systems to promote their own replication and evade the system's immune response. The onset of the viral infection triggers a series of changes in the host microenvironment to ensure the clearance of the pathogen and removal of infected cells. It is now thus well recognized that identification of host proteins of interest, after additional functional validation, may lead to new discoveries regarding host-virus interactions.

In the recent years proteomics- based studies have been significant in providing insights into how the host protein expression profile is altered during viral infection and hence fostered a new branch of study known as Viral Proteomics [2], [3]. Systems biology is another budding field of science which incorporates mathematical and bio-informatics tools to understand biological systems. There have been recent reports which analyze the viral proteomic data using systems biology in order to predict host- pathogen interactions [4]–[6].

Japanese encephalitis (JE), is one of the most prevalent forms of viral encephalitis worldwide, affecting mostly children and is responsible for around 15,000 deaths annually [7]. It is caused by a mosquito-borne flavivirus called Japanese encephalitis virus (JEV) which is a positive sense single stranded RNA virus that causes extensive neuronal death. JEV infection manifests with fever, headache, vomiting, signs of meningeal irritation and altered consciousness leading to high mortality and neurological sequelae in some of those who survive [8]. Recent reports have shown that this disease is spreading to other distant parts of the world, contrary to common belief that this disease is restricted to parts of Asia, thus increasing the need for a better understanding of JEV pathogenesis which may help in the development of different preventive strategies and new therapeutic interventions [9]. Proteomic study of host response to West Nile Virus (which is the nearest kin to JEV) infection in Vero cells has reported most of the altered host proteins to be involved in transcription/translation processes, alteration of the cytoskeleton networks, stress cellular response, and apoptotic pathways [10]. In a recent study of JEV infection in HeLa cells, Zhang et al. have reported IFITM3, RANBP2, SAMD9, and VAMP8 as potential antiviral factors with roles in JEV infection [3]. However, to the best of our knowledge, no such analysis has been carried out in any *in vivo* model for JEV infection.

Although a number of studies have been carried out to study the host response to viruses from the family flaviviridae, most of them have been limited to *in vitro* models which may not necessarily provide an insight into the actual host environment [10], [11]. This study has been designed to characterize the modifications induced in the host proteome by employing 2DE-MS approach in response to JEV infection in mouse model. In the present investigation we restricted our study to JEV-infection in neuronal cells. In total, 56 proteins were identified to be differentially expressed in brain and neurons. Most of the differentially regulated proteins were found to be associated with cellular metabolism followed by stress response, regulation of apoptosis, protein transport and localization and stabilizing cytoskeletal interactions. As we have used an *in vivo* mouse model in this study, the host response to JEV infection could be a cumulative effect of several factors including expression and transport of molecules from different tissue compartments, secretion, and/or post translational modifications.

In order to elucidate the interacting partners of the proteins differentially expressed in our proteomic analysis and establish a host-viral interactome model we applied bio-informatics tools. Bioinformatics analysis carried out using the NeuroDNet database for constructing and analyzing neurodegenerative disease associated gene networks revealed the interactions between the viral proteins and the host proteome (proteins which were differentially expressed in our case) and helped us to corroborate our findings.

## Materials and Methods

### Ethics Statement

All animal experiments were approved by the Institutional Animal and Ethics Committee of the National Brain Research Centre (approval no. NBRC/IAEC/2011/66). The animals were handled in strict accordance with good animal practice as defined by the Committee for the Purpose of Control and Supervision of Experiments on Animals, Ministry of Environment and Forestry, Government of India.

### Cells and viruses

**Cells** Mouse neuroblastoma (Neuro2a) purchased from National Centre For Cell Science, Pune, India and human neuroblastoma cell line (SHSY-5Y) (kind gift from Dr. Steve Levison, University of Medicine and Dentistry, New Jersey, USA, obtained from ATCC) were grown at 37°C in Dulbecco's modified Eagle medium (DMEM) supplemented with 3.7% sodium bi-carbonate (NaHCO_3_), 10% fetal bovine serum and penicillin/streptomycin. All the reagents related to cell culture were obtained from Sigma (St. Louis, USA), unless otherwise stated. Cells were maintained as monolayers in 10% CO_2_ and were passaged by trypsinization 2 to 3 times each week.

### Viruses

GP78 strain of JEV was propagated in suckling BALB/c mice and their brains were harvested when pathological symptoms were observed. Virus titrations were conducted and quantified as described earlier [12].

### JEV infection in mouse model

Adult BALB/c mice (4–6 weeks) were used in all experimental procedures. Mice were randomly assigned to two groups consisting of 4 animals each: Mock-infected control (Control) and JEV infected (JEV) (groups did not have sexual bias). Animals were injected with 3×10^5^ p.f.u. of JE virus of strain GP78 through tail vein while the control animals received phosphate buffered saline (PBS). Animals were sacrificed on fifth day post-infection when they started showing acute symptoms of JE including restriction of movements, limb paralysis, poor pain response, whole body tremor, pilo-erection, and hind limb paralysis. Tissues were snap frozen in liquid nitrogen and kept in −80°C till use.

### Infection of mouse neuroblastoma cell line with JE virus

Mouse neuroblastoma Neuro2a cells (Neuro2a) were plated at a density of 3×10^6^ cells per plate and cultured in serum containing media until cells reached 70–80% confluency. Cells were then either mock-infected or infected with JEV (4.4×10^9^ pfu/mL) at multiplicity of infection (MOI) of 5 for 1.5 h. Cells were then washed twice with sterile 1× PBS to remove non-internalized virus and were incubated for 36 h in serum free media.

### Protein extraction from brain tissue and neuroblastoma cell line

The brain tissues (∼0.4 g) were thawed on ice and homogenized using Ultra-turrax T8 homogenizer (IKA-Werke GmbH & Co. KG, Germany) in 500 µl solubilization buffer containing 8M urea, 2% (w/v) CHAPS, 0.2% sodium orthovanadate and 1× concentration of protease inhibitor cocktail for mammalian cells and tissue extracts (Sigma Aldrich, USA). The supernatant was collected by centrifugation and subjected to three pulses of sonication on ice followed by centrifugation at 20,000× g for 30 min at 4°C for the recovery of total soluble proteins. Protein extraction from Neuro2a cells was carried out in a similar manner. Protein concentration was quantified in supernatants by Bradford Protein Assay kit (Bio-Rad, USA) as per manufacturer's instructions and the extraction procedure was carried out according to [13].

### Two dimensional gel electrophoresis (2-DE)

2-DE was performed as described earlier [14]. The protein pellet was resuspended in sample rehydration buffer (8 M urea, 2% w/v CHAPS, 15 mM DTT and 0.5% v/v IPG buffer pH 3–10). The isoelectric focusing was performed using immobilized pH gradient (IPG) strips (Bio-Rad, USA). IPG strips of 7 cm size with a pH range from 4.0–7.0 and 5.0–8.0 were used for all the experiments. For the first dimension 500 µg of protein samples in 150 µl of rehydration solution was used to passively rehydrate IPG strips overnight. The proteins were then focused for 10000 VHr at 20°C under mineral oil on a Protean i12™ IEF Cell (Bio-Rad, USA). After focusing, the strips were incubated for 10 min, in 2 ml of equilibration buffer I (6 M urea, 30% w/v glycerol, 2% w/v SDS and 1% w/v DTT in 50 mM Tris/HCl buffer, pH 8.8) followed by equilibration buffer II (6 M urea, 30% w/v glycerol, 2% w/v SDS and 4% w/v iodoacetamide in 375 mM Tris/HCl buffer, pH 8.8). After the equilibration steps the strips were transferred to Tris glycine SDS-PAGE where proteins were separated on 10% and 14% acrylamide gels for the second dimension by the method of Blackshear [15].

### Protein visualization and image analysis

Protein spots were visualized by staining with Coomassie Brilliant Blue G-250. Gel images were captured by LI-COR odyssey infra-red imager (LI-COR Biosciences, USA). Four biological replicates each with two analytical replicate (n = 8) images per dataset (mock-infected control versus JEV infected) were used for automatic spot detection using PD Quest 2D Analysis Software (Hercules, CA, USA). Spot intensities were normalized by total valid spot intensities and mean of values from duplicate analytical gels from four biological replicates were subjected to paired *t*-test analysis using GraphPad Prism software. Protein spots showing altered expression between control and experimental groups (|ratio|≥1.5, *p*≥0.05) were marked and excised.

### In gel protein digestion

The in gel tryptic digestion of the protein spots was carried out as described previously [16]. Briefly, gel pieces excised from 2-DE gels were destained at room temperature with 200 µL 50%) ACN/50 mM NH_4_HCO_3_ for 1 h. The gel pieces were reduced (0.16% DTT in 50 mM NH_4_HCO_3_), alkylated (2% iodoacetamide in 50 mM NH_4_HCO_3_), dried and then digested with 100 ng trypsin (Sigma-Aldrich, USA) in 50 mM NH_4_HCO_3_ overnight at 37°C. Peptides were extracted with 50% acetonitrile and 0.1% trifluoro-acetic acid (TFA), dried, and resuspended in 0.5% TFA before MS analysis.

### Identification of protein spots by tandem mass spectrometry

Spots showing differential expression in brain and Neuro2a cells were analyzed at the central instrumental facility at the Delhi University South Campus and identified by Applied Biosystem 4800 plus MALDI TOF/TOF Analyzer (AB Sciex, USA) as previously described. For MALDI-TOF mass spectrometry, 1 µl of the digest was mixed with 2 µl of the matrix solution (α-cyano-4-hydroxycinnamic acid in 80% [vol/vol] acetonitrile and 0.1% [vol/vol] trifluoroacetic acid [TFA]), and 1 µl of this mixture was deposited onto the MALDI target. The spectrum was obtained in the mass range of 500 to 4,000 Da and was calibrated using a calibration mixture containing angiotensin I, Substance P, adrenocorticotropin(1–17) [ACTH(1–17)], ACTH(18–39), and somatostatin [17].Spectra were analyzed with MASCOT sequence-matching software from Matrix Science. The peak list was searched against the taxonomy group *Mus musculus* at non-redundant protein sequence database of NCBI with 144127 sequence entries. Search parameters were as follows: trypsin digestion with one missed cleavage, variable modifications (oxidation of methionine and carbamidomethylation of cysteine), and the peptide mass tolerance of 50 ppm for precursor ion and mass tolerance of ±0.6 Da for fragment ion with +1 charge state.

### Validation of proteomic results by Immuno-blot analysis

Total protein was extracted from brain tissues or mouse neuroblastoma cells (control and JEV infected) as described above. Protein concentration was quantified in supernatants by Bradford Protein Assay kit (Bio-Rad, USA) as per manufacturer's instructions. Equivalent amounts of the tissue lysates (50 µg) were separated on 10% SDS-PAGE and then transferred to a nitrocellulose membrane followed by blocking. The blots were incubated with respective primary antibodies (Abcam,Cambridge, UK) at 4°C overnight at dilutions recommended by the manufacturer as mentioned in Table S1. After extensive washes in PBST, the blots were incubated with appropriate (mouse or rabbit specific) secondary antibodies (1∶5000; Vector laboratories, USA). The blots were again rinsed in PBST and processed for development using an ECL Immuno-blot kit (Milipore, India). The images were captured and analyzed using Chemigenius Bioimaging System (Syngene, Cambridge, UK). The blots were stripped and reprobed with anti-β-tubulin (1∶1000; Santa Cruz Biotechnology, Santa Cruz, CA, USA) to determine equivalent loading of samples. Quantitative changes were determined by densitometric analysis of the blots.

### Immunostaining

Neuro2a cells were cultured in 4-well chamber slides and were either mock- infected or infected with JEV as described above. The cells were incubated for 36 h and then fixed in 4% paraformaldehyde followed by blocking with 5% BSA solution. The cells were then incubated overnight at 4°C with primary antibodies against JEV Nakayama strain (1∶100; Chemicon, USA). Following incubation, the cells were washed and incubated with FITC-conjugated secondary antibodies. Final washes were followed by mounting with 49-6- diamidino-2-phenylindole (DAPI, Vector laboratories Inc., CA, USA). Images were obtained using Zeiss Axioplan 2 upright digital microscope (Zeiss, Germany).

### Immunohistochemistry

JEV infected and age matched control BALB/c mice in sets of three were perfused transcardially with 1× PBS and whole brains isolated were fixed with 4% paraformaldehyde (PFA) in 1× PBS for 24 h at 4°C and subsequently kept in 30% sucrose for additional 24–48 h at 4°C. The brains were then processed for cryostat sectioning and the sections were stained for selected proteins and, a neuronal marker NeuN. Briefly, the cryostat sections were washed with 1× PBS and processed for antigen retrieval by incubating at 70°C for 1 h in antigen unmasking solution (Vector laboratories). The sections were then washed with 1× PBS and blocked for 1.5 h with 5% BSA in 1× PBS. Neurons were labeled with mouse anti-NeuN antibody (1∶500; Chemicon, USA), along with mouse/rabbit specific antibodies (1∶500; Abcam Cambridge, UK) by incubating the sections overnight at 4°C. After five washes with 1× PBS, the sections were incubated with horse anti-mouse Fluorescein Isothiocyanate (FITC, 1∶250; Vector Laboratories) for NeuN and goat anti-rabbit Alexa Fluor 594 (1∶1000; Molecular Probes, Oregon, USA) for selected antibodies for 1 h. The slides were then mounted with mounting medium containing DAPI (Vector laboratories). The images were captured with Zeiss Axioplan 2 upright digital microscope (40× magnification; Zeiss, Germany).

### Analysis of Protein-Protein Interaction Networks

For protein-protein interaction analysis, genes were annotated with homologous human genes using human genotype terms and codes (www.ncbi.nlm.nih.gov/genbank/). The interaction network for observed genes were obtained by using human protein interaction information provided by NeuroDNet [18] and visualized using Cytoscape [19]. The biological processes and biochemical pathways used in the analysis in this paper were obtained from the Gene Ontology (GO) [20], Kyoto Encyclopedia of Genes and Genomes (KEGG) [21] and Reactome [22] Pathway database. Statistically significant clusters from biological processes and biochemical pathways of genes into sub-graphs were identified using GO enrichment analysis plugin BiNGO [23] and Gene set enrichment analysis (GESA) [24] tool. Statistically significant clusters were found in the network by applying Hypergeometric test with Benjamini & Hochberg False Discovery Rate (FDR) correction [25]. The functional clusters of genes that were found to be significant in the experiments were used to construct a perturbed interaction network. The network consisted of viral and human protein interactions. The experimentally observed proteins overlapped with human protein interaction information with directional signal transduction and metabolic pathways. Based on the derived data, JEV pathogenesis network was reconstructed that shows several closely related genes networked with the genes found in the JEV infection analysis. The interaction information between JEV proteins and human proteins was obtained from the literature.

### Statistical analysis

The experimental data between two groups were analyzed using student's t-test, and data among several groups were analyzed by one-way ANOVA by GraphPad Prism 5 software. A *p* value<0.05 was considered significantly different. All results are presented as mean ± SD from at least three independent experiments, unless otherwise stated.

## Results

### Global host proteome response post- JEV infection *in vivo* and *in vitro* models

**Figure 1:**
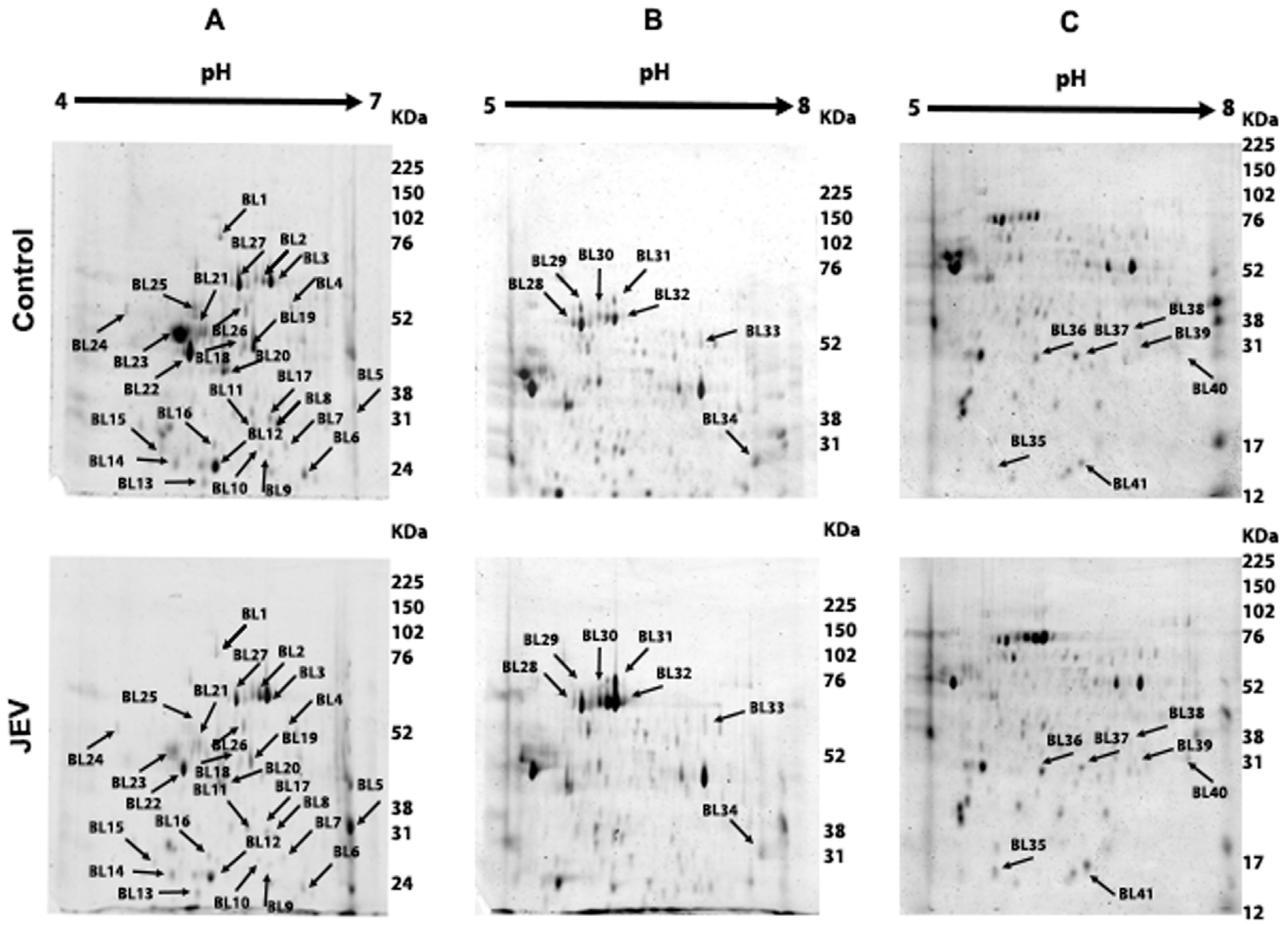
Proteomic Analysis of brain tissue lysate post-JEV infection. Equal amount of total protein from brain tissue lysates were separated on (**A**) an immobilized linear pH gradient IPG strips (4.0–7.0) (**B**) pH gradient IPG strips (5.0–8.0) and then in the second dimension on 10% SDS-PAGE and (**C**) 14% SDS-PAGE. Spots showing differential expression were marked and excised, and identified by MALDITOF/MS and database searches. The spots are labeled on the gel according to the numbers presented in Table S1.

**Figure 2:**
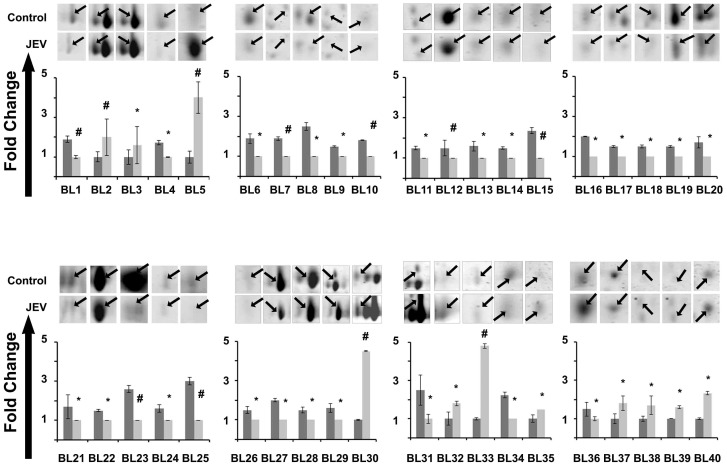
Relative fold change in differentially expressed proteins in brain tissue post-JEV infection. Spot intensities were normalized by total valid spot intensities and mean of values from duplicate analytical gels from four biological replicates were subjected to paired *t*-test analysis. Protein spots showing altered expression between control and experimental groups (|ratio|≥1.5, *p*<0.05) were marked and excised. Dark grey bars indicate spots from mock infected control sample while light grey bars indicate JEV infected sample. * = p<0.05, # = p<0.01. Data represented are means ± SD of four independent experiments.

**Figure 3.Differential:**
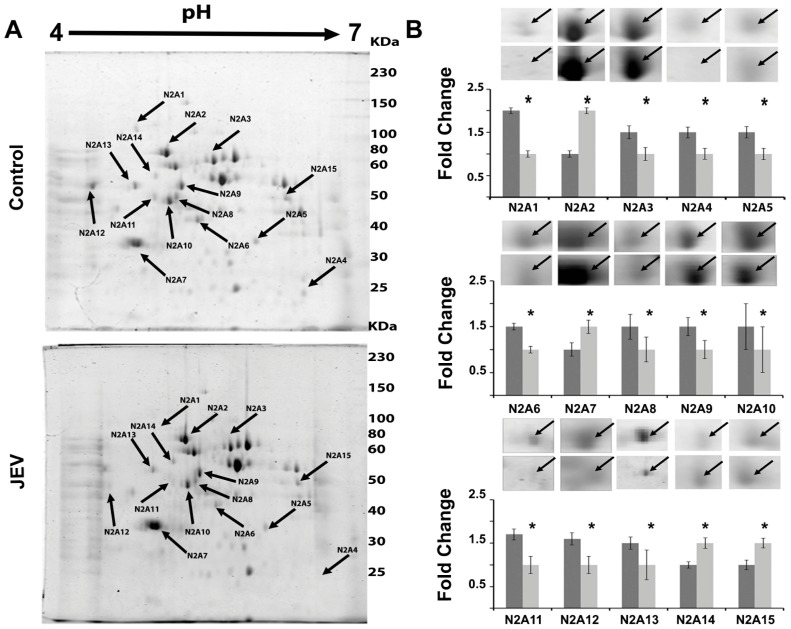
Differential protein expression in JEV infected Neuro2A cells. (**A**) A representative 2-DE gel of Neuroblastoma cell (Neuro2a) lysate from mock-infected and JEV infected cells. Equal amount of total protein from cell lysates were separated on an immobilized linear pH gradient IPG strips (4.0–7.0) and then in the second dimension on 10% SDS-PAGE. Spots showing differential expression were marked excised, and identified by MALDITOF/MS and database searches. The spots are labeled on the gel according to the numbers presented in table S1. (**B**) Protein spots representing relative fold change in expression in mouse neuroblastoma cell (Neuro2a) lysate.Spot intensities were normalized by total valid spot intensities and mean of values from duplicate analytical gels from four biological replicates were subjected to paired *t*-test analysis. Protein spots showing altered expression between control and experimental groups (|ratio|≥1.5, *p*<0.05) were marked and excised. Dark grey bars indicate spots from mock infected control sample while light grey bars indicate JEV infected sample. * = p<0.05. Data represented are means ± SD of four independent experiments.

JEV modulated host proteins were identified by proteomic analyses of JEV-infected mouse brain and JEV-infected Neuro2a cells comparing with their mock-infected controls. For brain tissues, the proteins were separated at different pH ranges of 4.0–7.0 and 5.0–8.0 and different SDS PAGE gel concentrations of 10 and 14% in order to ensure total coverage of the host proteome. For Neuro2a cells, the proteins were separated at pH 4.0–7.0. With the use of PD Quest software, 2-DE gel images for control versus JEV infected tissues were quantitatively analyzed as shown in Fig. 1, 2 & 3. A total of 165 and 51 protein spots were matched on 2-DE gels between control and treated brain and Neuro2a samples respectively. In brain total proteome 50 spots were found to be differentially regulated out of which 41 could be successfully identified. Among those, 11 spots were significantly up regulated (ratioJEV/control≥|1.5|, p<0.05, spots shown in Fig. 2) while the rest were found to be down regulated. Fifteen protein spots from Neuro2a showing significant changes in intensity ratio were observed. (4 spots were up-regulated and 11 spots were down-regulated, (ratioJEV/control≥|1.5|, p<0.05, spots shown in Fig. 3).

The observed MW and p*I* values of the protein spots on the 2- DE gels were compared with the theoretical MW and p*I* values of corresponding proteins. Most of the experimental values were found to be close to theoretical values, indicating unambiguous identification (Table S2). Certain differences in theoretical and experimental values were observed for some of the identified proteins which might be a result of post-translational proteolytic processing and modification whereas a difference in the p*I* values might be attributed to the cleavage of alkaline regions and phosphorylation of multiple residues.

### Differentially regulated proteins related to metabolism, protein folding and cytoskeleton post-JEV infection

All protein spots showing differential expression of 1.5 fold or greater (p<0.05) were excised, trypsin digested and identified by MALDITOF/TOF MS and MS/MS analysis. Table S2 corresponds to the spots in Fig. 1, 2 & 3 and lists the protein names; MOWSE score, matched peptides and sequence coverage of identified proteins; Mr and p*I* values; and fold changes between control and JEV infected group. Among the differentially expressed proteins in response to JEV infection, alfa fetoprotein, serum albumin precursor, and peroxiredoxin 6 isoforms were found to be the most abundant up regulated spots (Fig. 1, Table S2). In addition, brain tissue also showed over expression of aldolase C and proteasome subunit alpha type-1 while BiP, nucleobindin1 and heterogeneous nuclear ribonucleoprotein H were found to be over expressed in Neuro2a cells. Very few of the identified proteins which include gamma actin, calreticulin precursor and heat shock protein-8 were found common to both the brain lysate and Neuro2a cells which were used in the study to mimic host neuronal response to the neurotropic virus. We observed down regulation of various proteins which are involved in metabolism and protein transport (Table S2). The proteins identified could be functionally classified into various groups (http://ca.expasy.org/), including those involved in intracellular protein transport (VCP, CALR, ERP29 VIM, ACTG1, PRDX4, PDIA6), protein localization (ACTG1, AFP, CAPZB), stress induced response (STIP1, HSPA8, UCHL1, PSMA1, CCT5) and cellular metabolism (PDHB, PRDX6, LDHB, ALDOC, YWHAG, GAPDH, PRDX6, PDXP, CKB, ENO2), nuclear transport (NPM1, CALR, NUCB1), regulation of apoptosis (HSP90B1, HSPA5, HSPA8, CALR,ATP5B, IDH3A) and ER stress response (HSP90B1, HSPA5, HSPA8, CALR).

### Validation studies by Immuno-blot analysis and Immunohistochemistry

**Figure 4:**
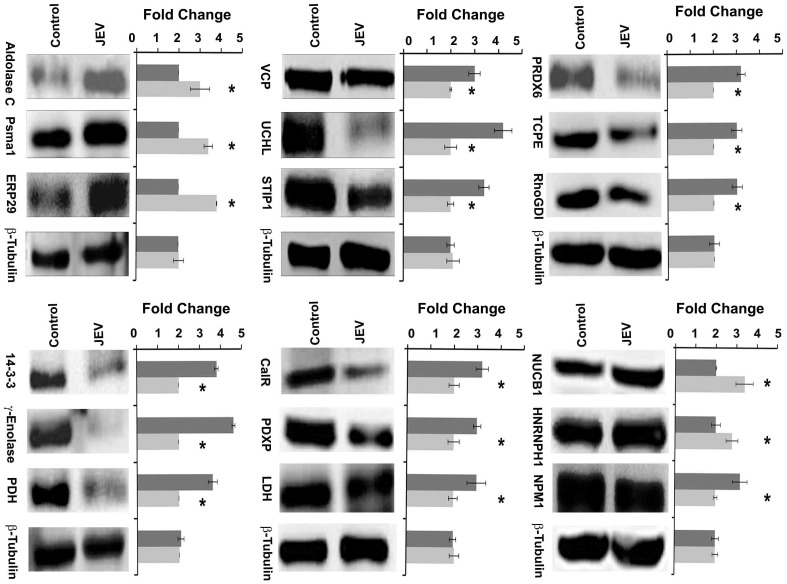
Validation of proteomic results using immunoblotting. Immunoblot of proteins showing altered expression in mouse brain with respect to mock-infected control. β- Tubulin was used as loading control. Dark grey bars indicate spots from mock infected control sample while light grey bars indicate JEV infected sample. Data represented are means ± SD of three independent experiments. * = p<0.05.

**Figure 5:**
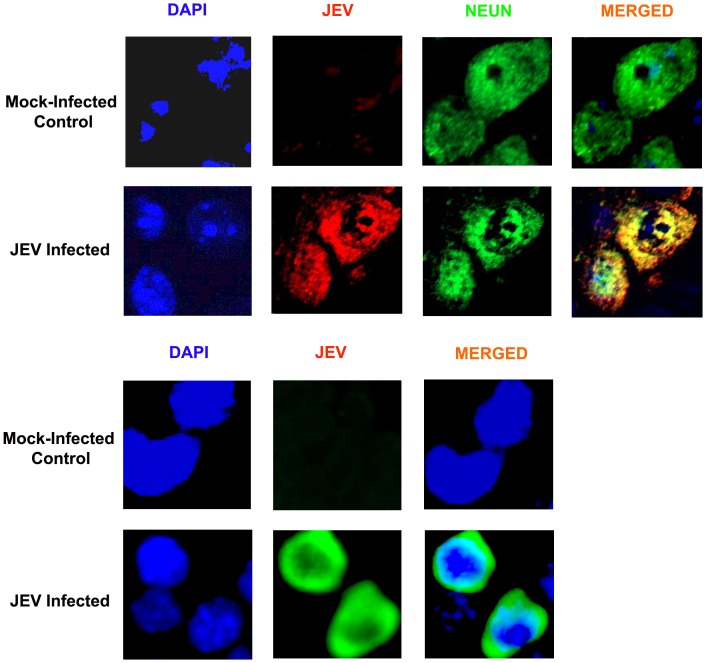
Confirmation of JEV infection in both *in vivo* and *in vitro* models using JEV specific antibody. The onset of JEV infection was confirmed by immunostaining of mouse brain sections and Neuro2a cells using JEV specific antibodies.

**Figure 6:**
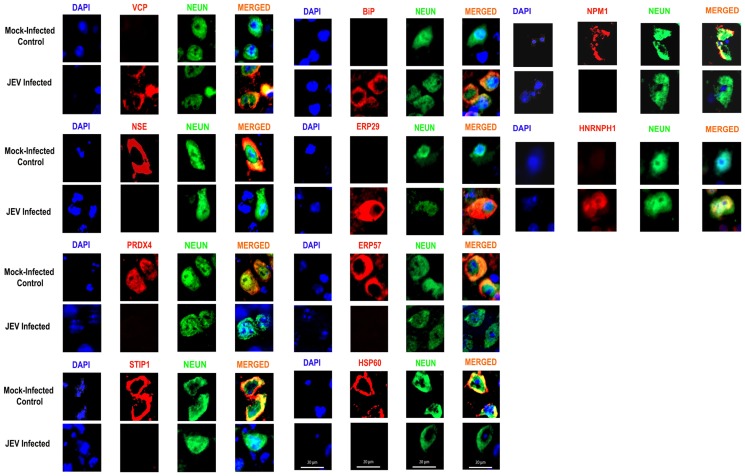
Validation of proteomic results using Immunostaining. Differential expression of selected proteins in neuron following JEV-infection in *in vivo* model. Images are representative of three independent experiments.

The proteomic results were validated by Immuno-blot analysis using specific commercially available antibodies against differentially expressed proteins. Immuno-blot data confirmed JEV induced over-expression of aldolase C, ERP29 and proteasome subunit alpha type-1 in brain tissue; and BiP, nucleobindin and heterogeneous nuclear ribonucleoprotein H over-expression in Neuro2a cells (Fig. 4). Antibody against the “housekeeping” protein, β tubulin was used as loading control to normalize the quantitative data of Immuno-blot analysis. The presence of virus in brains of JEV-infected animals as well as cells was confirmed by immunofluorescence using antibodies specific to JEV strain Nakayama (Fig. 5). The proteomic results were further reinforced with the help of immunohistochemistry using specific antibodies against randomly selected proteins belonging to different functional categories. The differential expression of the selected proteins in the cortical neurons could be distinctly visualized (Fig. 6). Immunostaining of brain from JEV-infected animals showed increased expression of ER stress associated proteins like ERP29 and BiP and decrease expression of NSE, PRDX4, STIP1, VCP, ERP57, and HSP60 that were co-localized with neuron-specific marker NeuN, as compared to control.

### The protein expression levels are modulated with disease progression

**Figure 7:**
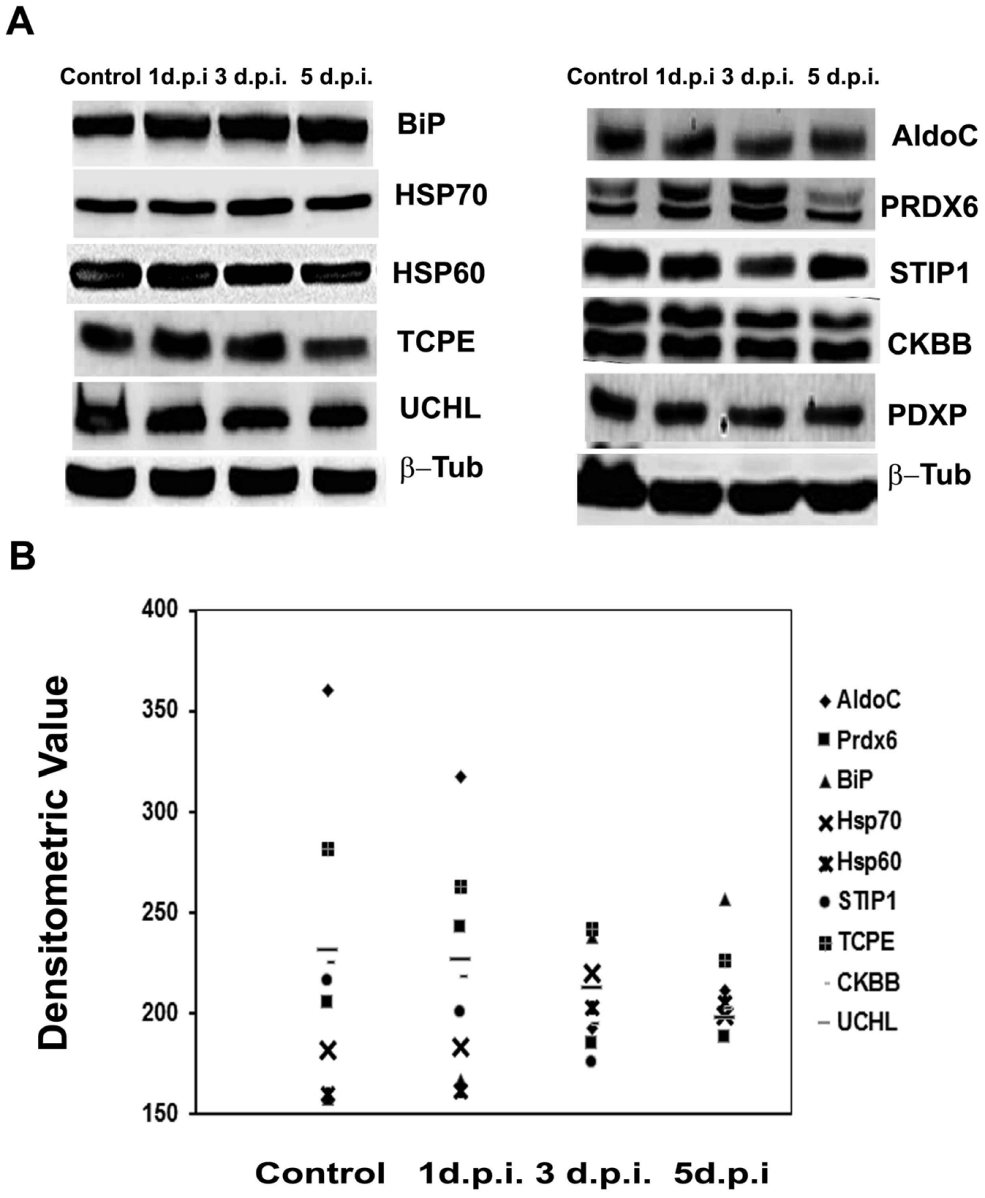
Temporal expression of selected proteins in mouse brain with disease progression. BALB/c mice of either sex were injected with 3×10^5^ p.f.u. of JE virus of strain GP78 through tail vein. The animals were then dissected at 1, 3, and 5 day post (JEV) infection (d.p.i) and brain was isolated for protein extraction. (**A**) Immunoblot showing the protein expression levels during JEV infectionas compared to that of mock-infected control mice. (**B**) Densitometric analysis of the differential protein expression. Data is representative of three independent experiments.

Immuno-blot analysis was carried out in order to study the expression of some selected proteins with the onset of infection (Fig. 7). The expression of proteins was studied on 0 (control), 1, 3, and 5 day post infection (d.p.i.). It was found that expression of proteins like BiP, HSP70, and HSP90 showed a gradual increase with the onset of infection. Peroxiredoxin 6 levels increased till third d.p.i. but thereafter showed a consistent decline. HSP60, stress induced phosphoprotein, T-complex protein 1 subunit epsilon, Creatine Kinase B, Pyridoxal phosphate phosphatase and ubiquitin carboxyl-terminal hydrolase PGP9.5 showed a consistent decline post infection.

### Proteome response to JEV infection in human neuronal cells correlates to the murine model

**Figure 8:**
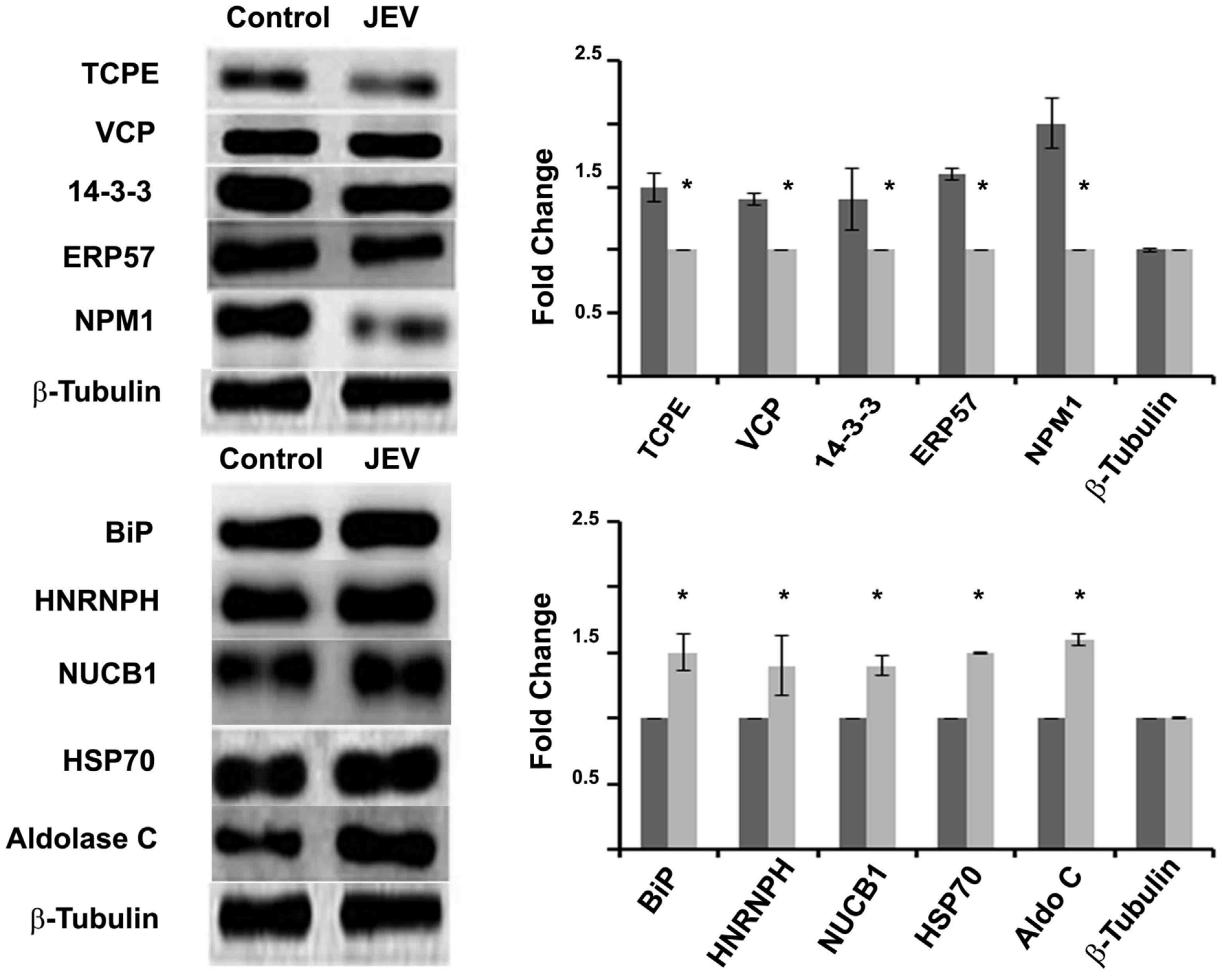
Expression of selected proteins in human neuroblastoma (SHSY 5Y) cells after JEV infection. SHSY 5Y cells were either mock-infected or infected with JEV (4.4×10^9^ pfu/mL) at multiplicity of infection (MOI) of 5 for 1.5 h. The expression of different proteins after JEV infection as compared to mock-infected controls was analyzed by immunoblotting. Data represented are means ± SD of three independent experiments. Dark grey bars indicate spots from mock infected control sample while light grey bars indicate JEV infected sample * = p<0.05.

Since Japanese encephalitis is a disease affecting the humans, we have tried to correlate the proteomic results obtained in a mouse model to that of humans. Immuno-blot analysis was carried out in human neuroblastoma cells SHSY 5Y to confirm whether a similar host response against JEV could be elicited in a human model (Fig. 8). It was found that our observations in the human model corroborates with that of our results in the mouse model. We found that the proteins, BiP, HNRNPH1, NUCB1, HSP70 and aldolase C were up regulated in response to JEV while TCPE, VCP, 14-3-3, ERP57 and nucleophosmin were down regulated.

### Analysis of the host protein-protein interaction involved in JEV infection

Based on our proteomic data, a systematic analysis of the host protein-protein interactions was carried out using NeuroDNet database. The proteins identified from the *in vivo* experiments were associated with intracellular protein transport (VCP, CALR, ERP29), protein localization (ACTG1, AFP, CAPZB), stress induced response (STIP1, HSPA8, UCHL1, PSMA1, CCT5) and cellular metabolism (PDHB, PRDX6, LDHB, ALDOC, YWHAG, GAPDH, PRDX6, PDXP, CKB, ENO2). These proteins were clustered in several pathways including Glycolysis/Gluconeogenesis, pyruvate metabolism, Citric Acid (TCA) cycle, destabilization of mRNA by AUF1 (hnRNP D0), ER Phagosome pathway, antigen processing-cross presentation, regulation of mRNA stability by proteins that Bind AU-rich elements. We expanded the protein interaction network and observed that the proteins were clustered in protein catabolism, ErbB1 downstream signaling, ubiquitination of a protein, insulin receptor pathway, mTOR signaling, ER-Phagosome pathway, assembly or disassembly of microtubules, E-cadherin signaling, signaling from the endoplasmic reticulum to the nucleus, ER-related stress response, induction of apoptosis, and oxidative stress response (Fig. S1, S2 & S6).

**Figure 9:**
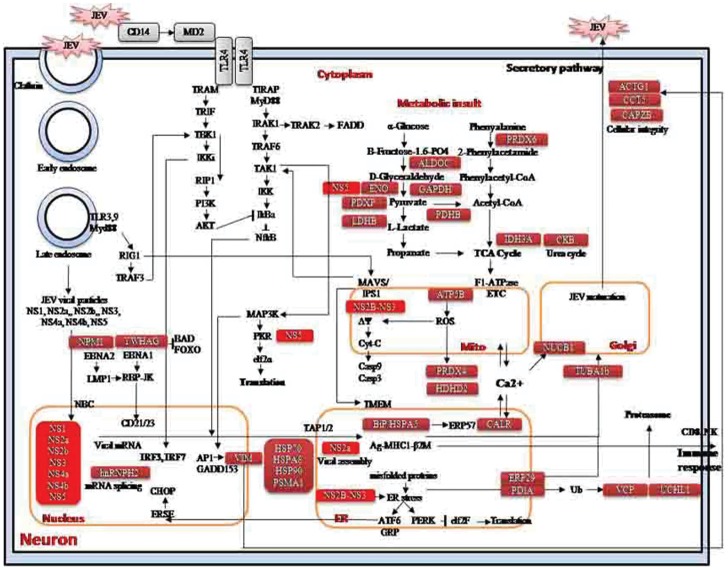
A schematic representation of the host response to JEV infection. Bioinformatic analysis of the proteins identified during the study was carried out to identify the various pathways and biological processes involved in the virus-host interaction. Based on the derived data, JEV pathogenesis network was constructed. Red tabs represent viral proteins and pink tabs represent the host proteins identified in this study.

The proteins identified during the *in vitro* experiments were found to be associated with nuclear transport (NPM1, CALR, NUCB1), regulation of apoptosis (HSP90B1, HSPA5, HSPA8, CALR, ATP5B, IDH3A), ER stress response (HSP90B1, HSPA5, HSPA8, CALR), and intracellular protein transport (VIM, ACTG1, PRDX4, PDIA6). These proteins were clustered in several pathways including activation of chaperones, unfolded protein synthesis, antigen processing and presentation, TCA cycle and respiratory electron transport, auroraB pathway. We expanded the protein interaction network and observed that the proteins were clustered in protein folding, insulin receptor signaling, endoplasmic reticulum to the nucleus signaling, upregulation of the activity of a caspase, a6b1 and a6b4 integrin signaling, p38 signaling mediated by MAPKAP kinases, TNFR1 signaling pathway, regulation of the actin cytoskeleton, involvement of mitochondria in apoptotic signaling, antigen processing and presentation, ER-related stress, assembly or disassembly of microtubules, protein sequestration and stabilization(Fig. S1, S2 &S6). Protein interaction network analysis (Fig. S3& S4) helped us to deduce the various interactions involved between the virus and the host during JEV infection thereby regulating various metabolic as well as apoptotic pathways. We found that the ER protein folding and secreting machinery was the most perturbed by this infection. Hence we also expanded the protein interaction network involved in the unfolded protein response and found *Hspa8*, *Hsp90b1*, *Hspa5*, *Pdia6*, *Calr* to be involved in unfolded protein response (UPR) initiated by endoplasmic reticulum stress (Fig. S5). Based on our analysis we proposed a mechanism of JEV infection and the possible changes in the host cellular machinery as a result. We deduced that during JEV infection the metabolic insult reduces the ATP level through glycolysis, TCA cycle and oxidative phosphorylation. The glucose sensors activate HSPA5 in ER and ROS in mitochondria induce stress response. The viral proteins inhibit the PERK and PKR response thus inducing the viral protein translation and reduce the level of host cytosolic proteins (Fig. 9). Table S3 shows the proteins selected for protein-protein interaction analysis.

## Discussion

Viruses exploit the host cellular machinery to complete their life cycle within their host. In our investigation an attempt has been made to decipher the neuropathogenesis of Japanese Encephalitis Virus (JEV) by studying the alterations made to the host proteome. We have applied 2DE-MS based approach to elucidate the proteomic changes in the host neurons post-JEV infection in a mouse model. Our study was aided with bioinformatic analysis to deduce the host- virus interactions and the various biological pathways affected from the time of viral entry into the neurons.

JEV deploys a clathrin independent mechanism to infect neuronal cells which requires dynamin and plasma membrane cholesterol [26]. After endocytosis, the incoming JEV particles are targeted to amphisomes for viral uncoating and an enhanced autophagy increases the amounts of entered viral RNA, leading to a higher level of viral protein expression and production. Enhanced autophagy has also been shown to negatively regulate the host innate antiviral response thus prolonging the viral progeny inside the host cells [27]. JEV then traffics through Rab5-positive early endosomes and release of the viral nucleocapsid occurs at the same stage. Various studies have proven that the flaviviral replication is not restricted to the cytoplasm but occurs in the host nucleus as well. There are strong evidences to show that the major replicase proteins NS3 and NS5 of JEV are localized within the nucleus during infection [28].

The viral core protein has been shown to co-localize with nucleolar protein nucleophosmin (NPM1) and cause its redistribution during infection suggesting a possible role in the intracellular localization of the core protein and replication of JEV [29]. NPM1 has been reported to function as a chaperone in the viral chromatin assembly process in infected cells [30]. Our investigation reports a decrease in the levels of NPM1 in JEV infected neurons which can be hypothesized as an attempt by the host neurons to arrest viral replication inside the cells. We also observed a remarkable increase in the protein heterogeneous nuclear ribonucleoprotein H (hnRNPH1) inside the neuronal cells which acts as a splicing factor involved in the regulation of rpL3 alternative splicing [31]. HNRNPH1 has also been implicated in assisting Hepatitis C virus replication [32]. Another protein HNRNPA2 a member of the same family has been reported to be involved in JEV replication [33]. These evidences suggest that the high level of HNRPH1 in the neurons is the resultant of the invasion of the host machinery by JEV in order to sustain its replication.

One of the pivotal weapons in the JEV arsenal is the cytoskeletal disorganization. The present research reinforces the phenomena by validating the alterations in various cytoskeletal proteins like f-actin capping protein, gamma actin and tubulin and vimentin post-JEV infection [34]–[37]. JEV infection is known to induce microtubular rearrangement and redistribution in Vero cells [38]. In addition to this the cytoskeletal perturbation observed during our investigation could also be a mode to facilitate the transport of viral proteins inside the cells through the rough endoplasmic reticulum (ER) and Golgi apparatus to the convoluted membrane, which may serve as a reservoir for viral proteins during JEV multiplication.

Previous reports claim targeting of mitochondria and alteration of metabolic pathways for the production of macromolecular substrates and energy during viral growth and replication. JEV infection induces mitochondrial disruption, reactive oxygen species [39] production and inflammation, which lead to apoptosis and cytopathology [40]. Peroxiredoxins have been shown to combat this oxidative insult by participating in antioxidant defense, redox signaling, apoptosis control and kinase modulation. Out of these, peroxiredoxin 6 (PRDX6) has been previously reported to be an important antioxidant enzyme in human brain defenses and has also been shown to play a neuroprotective role against hypoxia induced damage [41].The same study has also reported a decline in the levels of PRDX6 on prolonged exposure to ROS. These reports further bolster our claims showing an increase in the expression of PRDX 6 till 3 d.p.i. and a gradual decline in the levels with disease progression. Another member of the same family identified during the study in the Neuro2a lysate is peroxiredoxin 4 (PRDX4) which is an endoplasmic reticulum localized peroxiredoxin and is known to play an important role in disulphide formation and can be associated with ER stress response [42].

A tremendous assault on the host metabolic machinery was observed with a consistent decline in the levels of various metabolic enzymes like enolase, pyruvate dehydrogenase, lactate dehydrogenase, isocitrate dehydrogenase, glyceraldehyde 3 phosphate dehydrogenase (GAPDH) commonly involved in glycolysis and TCA cycle. Previous reports indicate that GAPDH interacts with 3′ ends of Japanese encephalitis virus RNA and co-localizes with the viral NS5 protein suggesting a possible role in viral replication [43]. Jaag et al. have shown that aldolase C is a multifunctional protein which apart from its metabolic role exhibits an endoribonuclease activity which may be associated with viral RNA degradation and recombination [44]. Our results have also shown a remarkable increase in aldolase C levels at later stages of infection which may be the host response to eliminate the viral replication. A member of the ATP synthase complex ATP5B was also found to be down-regulated and the decreased energy production as a result may also be a contributing factor in severe neurodegeneration [45]. Cytosolic brain specific creatine kinase expression was suppressed during JEV infection which may be one of the targets for reactive oxygen species in the brain in neurodegenerative disease [46].

ER is an essential organelle for viral replication and maturation as evidenced by previous reports. During the process of infection, a large amount of viral proteins are synthesized in infected cells, where the misfolded proteins induce the ER stress response which modulate various signaling pathways leading to cell survival or cell death [47]. The cellular machinery responds to this stress by induction of molecular chaperones, enhancement of degradation and clearance of unfolded proteins. This response is termed as unfolded protein response (UPR) [48]. An earlier study has shown that JEV infection triggers the UPR in fibroblast BHK-21 cells and in neuronal N18 and NT-2 cells, which results in apoptotic cell death [49]. Altered expression of chaperones such as glucose-regulated proteins (GRPs), BiP/GRP78 and other ER-resident proteins such as calreticulin and protein disulfide isomerase (PDI) can be associated with UPR [48] which corroborate our findings. We also observed an over expression of heat shock proteins HSP70 following JEV infection which may have a protective role against apoptosis [10]. Our investigation reports high levels of ERP29 which is an established member of the host stress response machinery and found to be associated with chaperones like BiP during UPR [50]. We observed a decrease in the levels of ERP57, a member of protein disulfide isomerase (PDI) family of foldases which is known to participate in signaling events from the plasma membrane as well as in regulatory events in the nucleus [51].We report a significant decline in the levels of calreticulin (CRT), an ER chaperone and play an important role in intracellular calcium homeostasis, which has been shown to interact with the NS5 protein of Dengue Virus. It has also been reported to have a possible role in flaviviral replication [52]. A decline in the levels of T complex protein subunit epsilon (CCT5), a chaperonin belonging to the HSP70 family of proteins was observed in the JEV infected mouse brain. Previous reports have shown this protein to be involved in interaction with Nonstructural protein NS5 of Hepatitis C virus nuclear antigen aiding its replication [51]. The down regulation of this protein may be a host defense mechanism to arrest the viral replication. Stress-induced phosphoprotein 1 (STIP1) also known as heat shock protein (HSP)-organizing protein levels were also found to be altered. Another important aspect of the UPR is the degradation of the misfolded proteins which involves the ubiquitin proteasome pathway. In the current study a down regulation of the proteins Ubiquitin C-terminal hydrolase-L1 (UCHL1) and Ubiquitin C-terminal hydrolase-L3 was observed. Neurodegeneration, posterior paralysis and dysphagia which are some of the key symptoms of JEV infection is associated with the loss of UCHL1 and UCHL3 [53]. Hence we may propose that the down regulation of these two proteins may play an important role in JEV associated pathologies but the underlying mechanism by which JEV brings about this alteration is not known. Valosin-containing protein (VCP) was found to be down regulated during JEV infection. Depletion or catalytic inhibition of VCP prevents capsid degradation and reduces neutralization by antibodies during host antiviral response which provides a possible explanation to our findings in our study [54]. Thus ER stress induced unfolded protein response (UPR), triggers a unique signaling cascade from the ER to the nucleus of a cell which activates expression of CHOP/GADD153, and appears to trigger activation of p38 mitogen-activated protein kinase, thus enhancing JEV-induced apoptosis [49].

In response to endoplasmic reticulum (ER) stress, an ER membrane-anchored transcription factor known as activating transcription factor 6 (ATF6), is transported to the Golgi apparatus and cleaved by site-1 protease (S1P) to activate the unfolded protein response (UPR) [55]. We observed an up-regulation of nucleobindin 1 (NUCB1) which is a golgi-localized negative feedback regulator in this ATF6-mediated branch of the UPR. Thus NUCB1 up regulation may suppress the unfolded protein response. The present study also reports an increase in the levels of the protein proteasome subunit alpha1 (Psma1) which is a subunit of the 20S proteasome or immunoproteasome involved in generating immunogenic peptides for presentation to CD8 T cells as part of immune surveillance. A recent study by Freudenburg et al. [56], has also shown that reduction in ATP levels lead to immunoproteasome activation during early antiviral response mediated by IFNβ in mouse pancreatic β-cells. This supports our claim of declining levels of the glycolytic enzymes in the JEV infected brain cells. A significant observation during our investigation was the ubiquitous serum albumin expression precursor in the brain. Recent studies have evidenced that albumin secretion is enhanced by microglial activation [57]. Microglial activation is a hallmark of JEV infection and these reports support the increase in the levels of albumin following JEV infection [58].

## Conclusions

In conclusion, this study provides a detailed proteomic analysis supported by bioinformatics to suggest that JEV invades the protein folding machinery of the host to ensure its replication and survival within the host brain thereby inducing ER stress leading to severe unfolded protein response. Apart from that oxidative stress, cytoskeletal disorganization and intracellular transport were also found to be affected during JEV infection. Our investigation has been a groundwork study in the field of neuropathogenesis post-JEV infection which will be of potential importance to investigators who will be interested in conducting anti-viral research in this field.

## Supporting Information

Figure S1 **Biological pathway enrichment analysis showing biological functions associated with proteins identified from** (**A**) Neuro2a cells (**B**) Mouse brain tissue. The red color nodes are the proteins and green nodes describes the functional biological process. The relation between nodes was shown by edges.
(TIF)

Figure S2 **Biological pathway enrichment analysis showing various pathways affected by the differentially regulation of the identified proteins during JEV infection** on (**A**) Neuro2a cells (**B**) Mouse brain tissue. The red color nodes are the proteins and green nodes describes the functional biological process. The relations between nodes were shown by edges.
(TIF)

Figure S3 **Protein interaction network involving differentially regulated proteins identified in the mouse brain.** The red color nodes are the proteins and green nodes describes the functional biological process. The relations between nodes were shown by edges.
(TIF)

Figure S4 **Protein interaction network involving differentially regulated proteins identified in the Neuro2a cells.** The red color nodes are the proteins and green nodes describes the functional biological process. The relations between nodes were shown by edges.
(TIF)

Figure S5 **The protein-protein interaction network of unfolded protein response** (UPR) associated genes encoding Erp29, Hsp90b1, Hspa5, Pdia6, Calr and HSPA8.
(TIF)

Figure S6 **Functional classification of the differentially affected proteins during JEV infection in** (**A**) mouse brain tissue (**B**) Neuro2a cells.
(TIF)

Table S1 **Antibody dilutions used for immunoblotting.**
(DOC)

Table S2 **Proteins showing differential expression after JEV infection, identified by MS/MS analysis of gel excised spots.**
(DOC)

Table S3 **List of identified proteins used for protein-protein interaction analysis.**
(DOC)
