# Supplementary figures and images for: Modulation of Neuronal Proteome Profile in Response to Japanese Encephalitis Virus Infection
Source: PLoS One. 2014 Mar 5;9(3):e90211. doi: 10.1371/journal.pone.0090211 (PMC3943924; doi:10.1371/journal.pone.0090211)

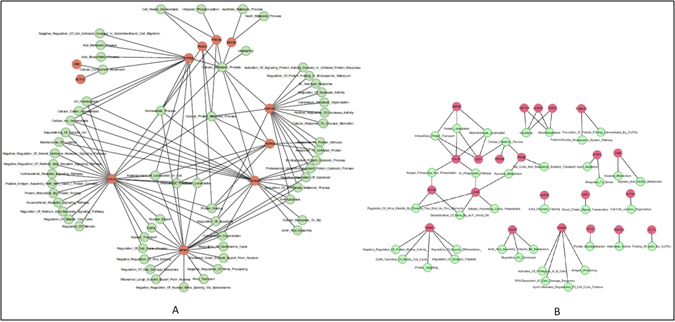

Supplement: Figure S1 — Biological pathway enrichment analysis showing biological functions associated with proteins identified from (A) Neuro2a cells (B) Mouse brain tissue. The red color nodes are the proteins and green nodes describes the functional biological process. The relation between nodes was shown by edges. (TIF) [file pone.0090211.s001.tif]

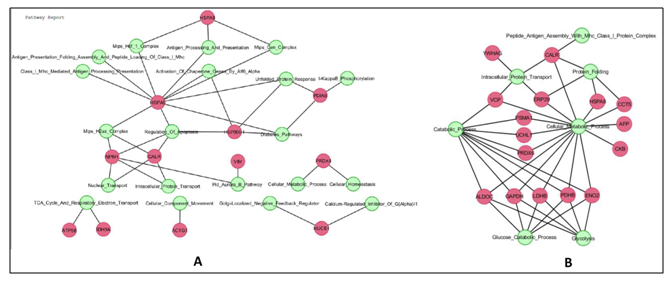

Supplement: Figure S2 — Biological pathway enrichment analysis showing various pathways affected by the differentially regulation of the identified proteins during JEV infection on (A) Neuro2a cells (B) Mouse brain tissue. The red color nodes are the proteins and green nodes describes the functional biological process. The relations between nodes were shown by edges. (TIF) [file pone.0090211.s002.tif]

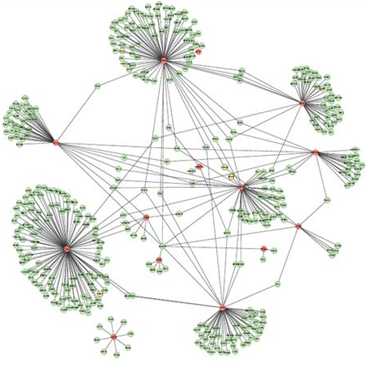

Supplement: Figure S3 — Protein interaction network involving differentially regulated proteins identified in the mouse brain. The red color nodes are the proteins and green nodes describes the functional biological process. The relations between nodes were shown by edges. (TIF) [file pone.0090211.s003.tif]

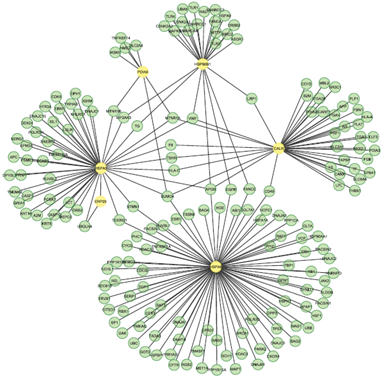

Supplement: Figure S4 — Protein interaction network involving differentially regulated proteins identified in the Neuro2a cells. The red color nodes are the proteins and green nodes describes the functional biological process. The relations between nodes were shown by edges. (TIF) [file pone.0090211.s004.tif]

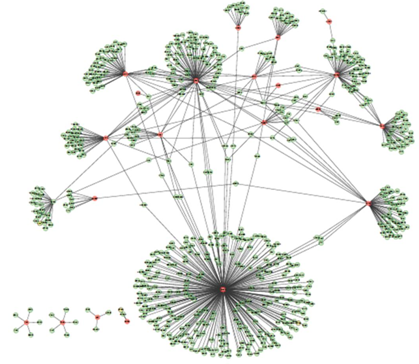

Supplement: Figure S5 — The protein-protein interaction network of unfolded protein response (UPR) associated genes encoding Erp29, Hsp90b1, Hspa5, Pdia6, Calr and HSPA8. (TIF) [file pone.0090211.s005.tif]

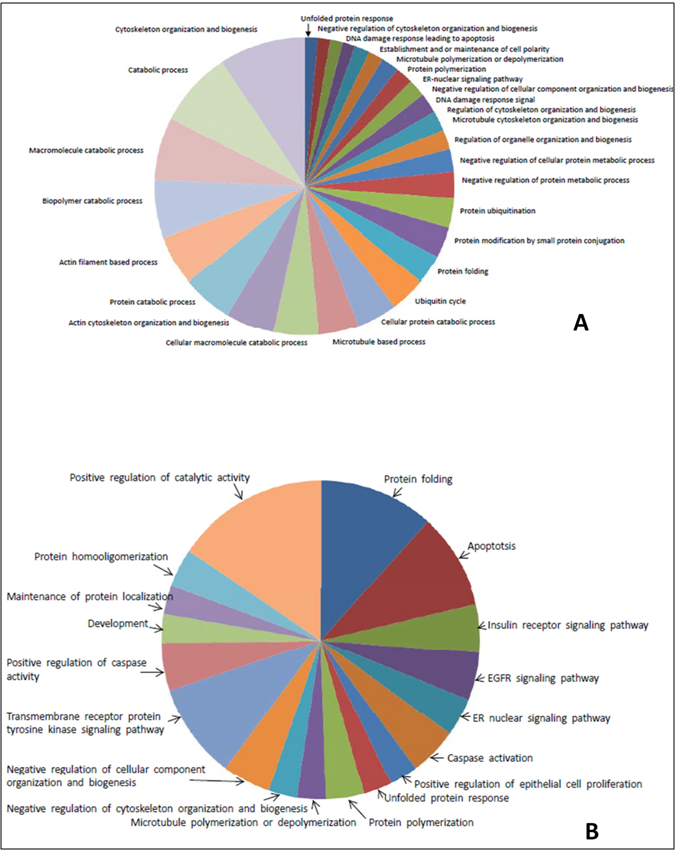

Supplement: Figure S6 — Functional classification of the differentially affected proteins during JEV infection in (A) mouse brain tissue (B) Neuro2a cells. (TIF) [file pone.0090211.s006.tif]
