# Supplementary material for: Modulation of Neuronal Proteome Profile in Response to Japanese Encephalitis Virus Infection
Source: PLoS One. 2014 Mar 5;9(3):e90211. doi: 10.1371/journal.pone.0090211 (PMC3943924; doi:10.1371/journal.pone.0090211)
Supplement: Table S1 — Antibody dilutions used for immunoblotting. (DOC) [file pone.0090211.s007.doc]

**Table S1. Proteins showing differential expression after JEV infection, identified by MS/MS analysis of gel excised spots.**

| **Sr. No** | **Spot No.** | **Protein ID.**  **(Accession)^a^** | **Matched peptides** | **Mowse Score^b^** | **Mp/Sc^c^** | **MW**  **(theor/ obs)** | **pI**  **(theor/obs)** | **Fold change^d^** |
| --- | --- | --- | --- | --- | --- | --- | --- | --- |
|  | BL1 | Transitional endoplasmic reticulum ATPase  (NP_033529) | R.GILLYGPPGTGK.T  R.EVDIGIPDATGRLEILQIHTK.N  R.ELQELVQYPVEHPDKFLK.F | 42 | 3/6 | 89293/81 | 5.14/5.5 | -1.9±0.17 |
|  | BL2 | Alpha fetoprotein  ( NP_001239590 ) | K.APQVSTPTLVEAAR.N  R.RHPDYSVSLLLR.L  K.LGEYGFQNAILVR.Y  K.DVFLGTFLYEYSR.R  R.LSQTFPNADFAEITK.L  R.YTQKAPQVSTPTLVEAAR.N | 442 | 6/16 | 47195/65.7 | 5.47/5.84 | 2±0.93 |
|  | BL3 | Alpha fetoprotein  ( NP_001239590) | K.APQVSTPTLVEAAR.N  R.RHPDYSVSLLLR.L  K.LGEYGFQNAILVR.Y  K.DVFLGTFLYEYSR.R  R.LSQTFPNADFAEITK.L  R.YTQKAPQVSTPTLVEAAR.N | 448 | 6/16 | 47195/65.7 | 5.47/5.85 | 1.6±0.93 |
|  | BL4 | T-complex protein 1 subunit epsilon  (NP_031663) | K.SQDDEIGDGTTGVVVLAGALLEEAEQLLDR.G  R.IADGYEQAAR.I  K.ISDKVLVDINNPEPLIQTAK.T  K.VLVDINNPEPLIQTAK.T  R.DVDFELIKVEGK.V  K.GVIVDKDFSHPQMPK.K  K.LDVMSVEDYKALQK.Y  R.WVGGPEIELIAIATGGR.I  R.FSELTSEKLGFAGVVQEISFGTTK.D  K.LGFAGVVQEISFGTTK.D  K.LGFAGVVQEISFGTTKDK.M  K.GSNDMQYQHVIETLIGKK.Q | 101 | 12/29 | 59586/ 59 | 5.72/6.12 | -1.7±0.12 |
|  | BL5 | Aldolase C, fructose-bisphosphate  ( NP_033787) | K.ISDRTPSALAILENANVLAR.Y  R.TPSALAILENANVLAR.Y  R.TVPPAVPGVTFLSGGQSEEEASLNLNAINR.C  R.ALQASALNAWR.G  R.DNAGAATEEFIKR.A | 448 | 5/20 | 39307/34 | 6.47/6.7 | 4±0.8 |
|  | BL6 | 1-Cys peroxiredoxin  (NP_031479) | M.PGGLLLGDEAPNFEANTTIGR.I  R.IRFHDFLGDSWGILFSHPR.D  R.FHDFLGDSWGILFSHPR.D  K.LPFPIIDDKGR.D  R.VVFIFGPDKK.L  K.LKLSILYPATTGR.N  K.LSILYPATTGR.N | 402 | 7/33 | 24855/24.7 | 5.71/6.2 | -1.9±0.224 |
|  | BL7 | haloacid dehalogenase-like hydrolase domain-containing protein 2 isoform 1  (NP_084102) | K.AVLVDLNGTLHIEDAAVPGAQEALKR.L  K.KLEFEISEDEIFTSLTAAR.N  K.TFFLEALR.D | 273 | 3/20 | 28712/27.8 | 5.70/6.1 | -1.9±0.09 |
|  | BL8 | L-lactate dehydrogenase B chain  (NP_032518) | K.IVVVTAGVR.Q  K.IVVVTAGVRQQEGESR.  R.QQEGESRLNLVQR.N  R.NVNVFKFIIPQIVK.Y  K.FIIPQIVK.Y  R.FRYLMAEK.L  K.LKDDEVAQLR.K  K.DDEVAQLRK.S | 90 | 8/16 | 36589/31.0 | 5.70/5.9 | -2.5±0.19 |
|  | BL9 | F-actin-capping protein subunit beta  ( NP_001032850) | -.MSDQQLDCALDLMR.R  R.RLPPQQIEK.N  R.RLPPQQIEK.N  R.KLEVEANNAFDQYR.D  K.SGSGTMNLGGSLTR.Q  R.LVEDMENKIR.S  K.IRSTLNEIYFGK.T  R.STLNEIYFGK.T  R.STLNEIYFGKTK.D | 59 | 8/26 | 31326/27.1 | 5.47/5.85 | -1.5±0.04 |
|  | BL10 | Pyridoxal phosphate phosphatase  (NP_064667) | R.IVPGAPELLQR.L  R.LSGPPDASGAVFVLGGEGLR.A  R.AVLVGYDEQFSFSR.L | 118 | 3/15 | 31492/27.1 | 5.53/5.84 | -1.8±0.14 |
|  | BL11 | Pyruvate dehydrogenase chain B protein  ( NP_077183 ) | R.IMEGPAFNFLDAPAVR.V  K.TYYMSAGLQPVPIVFR.G | 66 | 2/10 | 34814/30.3 | 5.63/5.8 | -1.5±0.08 |
|  | BL12 | ubiquitin carboxyl-terminal hydrolase PGP9.5  ( NP_035800) | K.LGVAGQWR.F  K.QIEELKGQEVSPK.V  K.QFLSETEKLSPEDR.A  R.VDDKVNFHFILFNNVDGHLYELDGR.M  R.MPFPVNHGASSEDSLLQDAAK.V  R.EFTEREQGEVR.F | 574 | 6/41 | 24822/26.1 | 5.33/5.45 | -1.5±0.38 |
|  | BL13 | Rho GDP dissociation inhibitor (GDI) alpha  (NP_598557) | R.VAVSADPNVPNVIVTR.L  K.QSFVLKEGVEYR.I | 86 | 2/13 | 23377/ 24 | 5.12/5.3 | -1.6±0.23 |
|  | BL14 | ubiquitin carboxyl-terminal hydrolase isozyme L3  (NP_057932) | R.WLPLEANPEVTNQFLK.Q  K.MHFESGSTLKK.F  R.AKFLENYDAIR.V  K.FLENYDAIR.V  R.VTHETSAHEGQTEAPSIDEK.V  K.FMERDPDELR.F | 68 | 7/35 | 26135/26.1 | 4.96/5.2 | -1.5±0.06 |
|  | BL15 | 14-3-3 protein gamma  (NP_061359) | K.NVTELNEPLSNEER.N  R.YLAEVATGEKR.A | 50 | 2/10 | 28345/27.8 | 4.80/5.1 | -2.36±0.14 |
|  | BL16 | Glyoxalase domain-containing protein 4  (NP_080305) | K.VAEGIFETEAPGGYKFYLQDR.S  K.IYEQDEEKQR.A  R.ALLGYADNQCKLELQGIQGAVDHAAAFGR.I | 76 | 3/20 | 33296/31.0 | 5.28/5.45 | -2±0.02 |
|  | BL17 | F-actin-capping protein subunit alpha-2  ( NP_031630.1) | K.FIIHAPPGEFNEVFNDVR.L  R.LLLNNDNLLR.E | 48 | 2/9 | 32947/36.6 | 5.57/5.85 | -1.5±0.05 |
|  | BL18 | Creatine kinase B-type  (NP_067248) | K.VLTPELYAELR.A  K.LAVEALSSLDGDLSGR.Y  K.TFLVWINEEDHLR.V  R.GTGGVDTAAVGGVFDVSNADR.L | 173 | 4/16 | 42686/50.6 | 5.40/5.7 | -1.5±0.08 |
|  | BL19 | Creatine kinase B-type  (NP_067248) | M.PFSNSHNTQK.L  K.VLTPELYAELR.A  K.LAVEALSSLDGDLSGR.Y  R.GIWHNDNKTFLVWINEEDHLR.V  K.TFLVWINEEDHLR.V  R.GTGGVDTAAVGGVFDVSNADR.L | 287 | 6/20 | 42686/50.6 | 5.40/5.8 | -1.5±0.04 |
|  | BL20 | Gamma-actin  ( NP_033739) | K.AGFAGDDAPR.A  R.AVFPSIVGRPR.H  R.VAPEEHPVLLTEAPLNPK.A  R.GYSFTTTAER.E  K.SYELPDGQVITIGNER.F  K.QEYDESGPSIVHR.K | 289 | 6/21 | 40992/45.0 | 5.56/5.5 | -1.7±0.27 |
|  | BL21 | Creatine kinase B-type  (NP_067248) | K.LRFPAEDEFPDLSSHNNHMAK.V  K.VLTPELYAELR.A  R.HGGYQPSDEHKTDLNPDNLQGGDDLDPNYVLSSR.V  K.LAVEALSSLDGDLSGR.Y  R.GIWHNDNKTFLVWINEEDHLR.V  K.TFLVWINEEDHLR.V | 560 | 6/27 | 42686/52.0 | 5.40/5.3 | -1.7±0.6 |
|  | BL22 | Gamma-enolase  (NP_038537) | R.AAVPSGASTGIYEALELR.D  K.AGAAERDLPLYR.H  K.LAMQEFMILPVGAESFR.D  R.LGAEVYHTLK.G  K.MVIGMDVAASEFYR.D  R.YITGDQLGALYQDFVR.N  R.IEEELGDEAR.F  R.FAGHNFRNPSVL.- | 585 | 8/25 | 47267/49.2 | 4.99/5.22 | -1.5±0.07 |
|  | BL23 | Tubulin beta-2B chain  ( NP_076205) | R.FPGQLNADLR.K  R.FPGQLNADLRK.L  K.LAVNMVPFPR.L  R.LHFFMPGFAPLTSR.G  R.YLTVAAIFR.G  K.TAVCDIPPR.G  R.ISEQFTAMFR.R | 406 | 7/14 | 49921/52.0 | 4.78/5.15 | -2.6±0.17 |
|  | BL24 | Gamma-enolase  (NP_038537) | K.AGAAERDLPLYR.H  K.LAMQEFMILPVGAESFR.D  K.MVIGMDVAASEFYR.D  R.YITGDQLGALYQDFVR.N  R.FAGHNFRNPSVL.- | 388 | 6/16 | 47267/55.0 | 4.99/4.65 | -1.6±0.20 |
|  | BL25 | Alpha-tubulin isotype M-alpha-2  (NP_035783) | R.AVFVDLEPTVIDEVR.T | 42 | 1/3 | 50134/55.0 | 4.94/5.25 | -3±0.20 |
|  | BL26 | Calreticulin precursor  (NP_031617) | K.EQFLDGDAWTNRWVESK.H  K.FEPFSNK.G  K.DMHGDSEYNIMFGPDICGPGTK.K  K.KVHVIFNYK.G  K.VHVIFNYKGK.N  K.NVLINKDIR.S  K.IDNSQVESGSLEDDWDFLPPKK.I  K.IKDPDAAKPEDWDER.A | 66 |  | 38064/54.0 | 4.91/5.7 | -1.5±0.18 |
|  | BL27 | Heat shock protein 8  ( NP_112442) | R.TTPSYVAFTDTER.L  K.TVTNAVVTVPAYFNDSQR.Q  K.DAGTIAGLNVLR.I  R.IINEPTAAAIAYGLDKK.V  K.STAGDTHLGGEDFDNR.M  R.TLSSSTQASIEIDSLYEGIDFYTSITR.A  K.LDKSQIHDIVLVGGSTR.I  K.LLQDFFNGKELNK.S  K.QTQTFTTYSDNQPGVLIQVYEGER.A | 637 | 9/24 | 70828/58.0 | 5.28/5.7 | -2±0.09 |
|  | BL28 | Heat shock protein 8  ( NP_112442) | R.TTPSYVAFTDTER.L  K.TVTNAVVTVPAYFNDSQR.Q  K.DAGTIAGLNVLR.I  R.IINEPTAAAIAYGLDKK.V  K.STAGDTHLGGEDFDNR.M  R.TLSSSTQASIEIDSLYEGIDFYTSITR.A  R.FEELNADLFR.G  K.LDKSQIHDIVLVGGSTR.I  K.QTQTFTTYSDNQPGVLIQVYEGER.A | 366 | 9/23 | 70828/57.6 | 5.28/5.9 | -1.5±0.15 |
|  | BL29 | Heat shock cognate 71 kDa protein  (NP_112442) | R.TTPSYVAFTDTER.L  K.TVTNAVVTVPAYFNDSQR.Q  K.STAGDTHLGGEDFDNR.M  K.LDKSQIHDIVLVGGSTR.I | 68 | 4/9 | 70793/60.4 | 5.37/5.9 | -1.6±0.22 |
|  | BL30 | Serum albumin precursor  (NP_033784) | R.YNDLGEQHFK.G  K.APQVSTPTLVEAAR.N  R.RHPDYSVSLLLR.L  K.LGEYGFQNAILVR.Y  K.DVFLGTFLYEYSR.R  R.LSQTFPNADFAEITK.L  R.RPCFSALTVDETYVPK.E  K.AADKDTCFSTEGPNLVTR.C | 492 | 8/18 | 68648/57.6 | 5.75/6.1 | 4.5±0.02 |
|  | BL31 | Serum albumin precursor  (NP_033784) | R.YNDLGEQHFK.G  R.LSQTFPNADFAEITK.L  K.DVFLGTFLYEYSR.R  R.RHPDYSVSLLLR.L  K.LGEYGFQNAILVR.Y  K.APQVSTPTLVEAAR.N | 334 | 6/12 | 68648/61.8 | 5.75/6.2 | -2.5±0.79 |
|  | BL32 | Alpha feto protein  ( NP_001239590) | R.LSQTFPNADFAEITK.L  K.DVFLGTFLYEYSR.R  R.RHPDYSVSLLLR.L  K.LGEYGFQNAILVR.Y  R.YTQKAPQVSTPTLVEAAR.N  K.APQVSTPTLVEAAR.N | 387 | 6/16 | 47195/57.6 | 5.47/6.2 | 1.8±0.34 |
|  | BL33 | Serum albumin precursor  (NP_033784) | R.EAHKSEIAHR.Y  R.YNDLGEQHFKGLVLIAFSQYLQK.C  K.ENPTTFMGHYLHEVAR.R  R.AFKAWAVAR.L  R.LSQTFPNADFAEITK.L  R.LSQTFPNADFAEITKLATDLTK.V  K.NYAEAKDVFLGTFLYEYSR.R  K.DVFLGTFLYEYSR.R  K.DVFLGTFLYEYSRR.H  R.RHPDYSVSLLLR.L  R.HPDYSVSLLLR.L  K.LGEYGFQNAILVR.Y  R.YTQKAPQVSTPTLVEAAR.N  K.APQVSTPTLVEAAR.N | 110 | 14/23 | 68648/53.4 | 5.75/6.9 | 4.8±0.07 |
|  | BL34 | Glyceraldehyde-3-phosphate dehydrogenase  (NP_032110) | K.VIHDNFGIVEGLMTTVHAITATQK.T  K.LISWYDNEYGYSNR.V | 93 | 2/11 | 35787/31.0 | 8.44/7.45 | -2.24±0.15 |
|  | BL35 | Stress-induced-phosphoprotein 1  (NP_058017 ) | K.LDPQNHVLYSNRSAAYAK.K  K.TVDLKPDWGKGYSR.K  K.FMNPFNLPNLYQKLENDPR.T  R.SLLSDPTYRELIEQLQNKPSDLGTK.L  R.IGNSYFKEEK.Y  K.YKDAIHFYNK.S  K.YKDAIHFYNK.S  K.ILKEQER.L  R.LAYINPDLALEEK.N  K.HYTEAIKR.N  K.LMDVGLIAIR.- | 67 | 11/26 | 62542/13.4 | 6.40/5.8 | 1.5±0.19 |
|  | BL36 | Peroxiredoxin-6  ( NP_031479) | M.PGGLLLGDEAPNFEANTTIGR.I  R.IRFHDFLGDSWGILFSHPR.D  R.FHDFLGDSWGILFSHPR.D  R.VVFIFGPDKK.L  K.LSILYPATTGR.N | 158 | 5/27 | 24855/26.0 | 5.71/6.2 | -1.5±0.36 |
|  | BL37 | Peroxiredoxin-6  ( NP_031479) | M.PGGLLLGDEAPNFEANTTIGR.I  R.IRFHDFLGDSWGILFSHPR.D  R.DFTPVCTTELGR.A + Carbamidomethyl (C)  R.VVFIFGPDKK.L  K.LKLSILYPATTGR.N  K.LSILYPATTGR.N | 320 | 6/33 | 24811/26.0 | 5.98/6.52 | 1.8±0.38 |
|  | BL38 | 1-Cys peroxiredoxin protein  (NP_031479) | M.PGGLLLGDEAPNFEANTTIGR.I  K.LAPEFAKR.N  R.VVFIFGPDKK.L  K.LSILYPATTGR.N  R.NFDEILR.V | 288 | 5/25 | 24825/29.0 | 5.98/6.8 | 1.7±0.49 |
|  | BL39 | Peroxiredoxin-6  (NP_031479) | M.PGGLLLGDEAPNFEANTTIGR.I  R.IRFHDFLGDSWGILFSHPR.D  K.LAPEFAKR.N  R.VVFIFGPDKK.L  K.LKLSILYPATTGR.N  K.LSILYPATTGR.N  R.NFDEILR.V  R.VVDSLQLTGTKPVATPVDWK.K | 483 | 8/43 | 24811/28.0 | 5.98/6.82 | 1.6±0.08 |
|  | BL40 | Proteasome subunit alpha type-1  (NP_036095) | R.NQYDNDVTVWSPQGR.I  R.IHQIEYAMEAVKQGSATVGLK.S  K.THAVLVALKR.A  K.KILHVDNHIGISIAGLTADAR.L  K.ILHVDNHIGISIAGLTADAR.L  R.FVFDRPLPVSR.L  R.LVSLIGSKTQIPTQR.Y  R.SQSARTYLER.H  R.ALRETLPAEQDLTTK.N | 106 | 9/44 | 29528/28.0 | 6.00/6.82 | 1.5±0.18 |
|  | BL41 | Endoplasmic reticulum protein 29, isoform CRA_a  (NP_080405) | K.LDKESYPVFYLFR.D  K.ESYPVFYLFR.D  K.WASQYLK.I  K.WASQYLKIMGK.I  K.IMGKILDQGEDFPASEMAR.I  K.ILDQGEDFPASEMAR.I  K.SLNILTAFR.K | 81 | 8/30 | 17858/14.1 | 7.68/7.6 | 2.38±0.09 |
| 42 | N2a1 | Heat shock protein 90, beta (Grp94), member 1    (NP_035761) | K.SEKFAFQAEVNR.M  K.FAFQAEVNR.M  R.ELISNASDALDKIR.L  R.LISLTDENALAGNEELTVK.I  K.EEASDYLELDTIKNLVR.K  K.SILFVPTSAPR.G  R.GLFDEYGSK.K  R.RVFITDDFHDMMPK.Y  K.YLNFVKGVVDSDDLPLNVSR.E  K.GVVDSDDLPLNVSR.E  K.LGVIEDHSNRTR.L  R.KEAESSPFVER.L  K.EAESSPFVERLLK.K  K.EFEPLLNWMKDK.A  K.KTFEINPR.H  R.HPLIRDMLR.R  K.AYGDRIER.M | 124 | 20/22 | 92432/90 | 4.74/4.8 | -2±0.07 |
| 43 | N2a2 | BiP  ( NP_071705) | R.ITPSYVAFTPEGER.L  R.ITPSYVAFTPEGERLIGDAAK.N  K.NQLTSNPENTVFDAKR.L  K.VTHAVVTVPAYFNDAQR.Q  R.IINEPTAAAIAYGLDKR.E  R.IEIESFFEGEDFSETLTR.A  R.AKFEELNMDLFR.S  K.DNHLLGTFDLTGIPPAPR.G  R.GVPQIEVTFEIDVNGILR.V | 1158 | 9/20 | 72433/70 | 5.10/5.15 | -1.5±0.07 |
| 44 | N2a3 | Heat shock protein 8  ( NP_112442) | K.NQVAMNPTNTVFDAKR.L  K.TVTNAVVTVPAYFNDSQR.Q  K.DAGTIAGLNVLR.I  R.IINEPTAAAIAYGLDKK.V  K.STAGDTHLGGEDFDNR.M  R.TLSSSTQASIEIDSLYEGIDFYTSITR.A  R.ARFEELNADLFR.G  K.LDKSQIHDIVLVGGSTR.I  K.QTQTFTTYSDNQPGVLIQVYEGER.A | 1258 | 9/24 | 70828/60 | 5.28/5.6 | -1.5±0.15 |
| 45 | N2a4 | Peroxiredoxin-4 precursor  (NP_058044) | K.DYGVYLEDSGHTLR.G  R.GLFIIDDKGVLR.Q  R.QITLNDLPVGR.S | 90 | 3/13 | 31033/25 | 6.67/6.6 | -1.5±0.12 |
| 46 | N2a5 | Isocitrate dehydrogenase [NAD] subunit alpha, mitochondrial precursor  (NP_083849) | K.IFDAAKAPIQWEER.N  K.APIQWEER.N  K.TPIAAGHPSMNLLLR.K  R.IAEFAFEYAR.N | 240 | 4/10 | 39613/37 | 6.27/6.06 | -1.5±0.13 |
| 47 | N2a6 | Gamma-actin  ( NP_033739) | K.AGFAGDDAPR.A  R.AVFPSIVGRPR.H  R.VAPEEHPVLLTEAPLNPK.A  R.TTGIVMDSGDGVTHTVPIYEGYALPHAILR.L  K.SYELPDGQVITIGNER.F  K.IIAPPERK.Y  K.QEYDESGPSIVHR.K | 468 | 7/28 | 40992/39 | 5.56/5.48 | -1.5±0.31 |
| 48 | N2a7 | Nucleophosmin isoform 1  (NP_032748) | K.MSVQPTVSLGGFEITPPVVLR.L  R.MTDQEAIQDLWQWR.K  R.MTDQEAIQDLWQWRK.S | 366 | 3/12 | 32540/32 | 4.62/4.7 | 1.5±0.14 |
| 49 | N2a8 | Protein disulfide isomerase associated 6  ( NP_082235) | K.LAAVDATVNQVLASR.Y  K.IFQKGESPVDYDGGR.T  K.GSFSEQGINEFLR.E  R.GSTAPVGGGSFPTITPR.E | 282 | 4/13 | 48627/47 | 5.05/5.22 | -1.5±0.32 |
| 50 | N2a9 | Vimentin  (NP_035831) | R.TYSLGSALRPSTSR.S  R.SLYSSSPGGAYVTR.S  R.LLQDSVDFSLADAINTEFKNTR.T  K.ILLAELEQLKGQGK.S  K.FADLSEAANRNNDALR.Q  R.ISLPLPTFSSLNLR.E | 618 | 6/20 | 53689/50 | 5.06/5.24 | -1.5±0.4 |
| 51 | N2a10 | Atp5b protein  (NP_058054) | R.LVLEVAQHLGESTVR.T  K.VLDSGAPIKIPVGPETLGR.I  K.AHGGYSVFAGVGER.T  K.VALVYGQMNEPPGAR.A  R.FTQAGSEVSALLGR.I  R.IPSAVGYQPTLATDMGTMQER.I  R.AIAELGIYPAVDPLDSTSR.I  R.IMDPNIVGNEHYDVAR.G  K.SLQDIIAILGMDELSEEDKLTVSR.A | 1244 | 9/29 | 56632/45 | 5.24/5.18 | -1.5±0.34 |
| 52 | N2a11 | Tubulin beta-5 chain  (NP_035785) | R.ISVYYNEATGGKYVPR.A  K.LAVNMVPFPR.L  R.LHFFMPGFAPLTSR.G  R.YLTVAAVFR.G  R.ISEQFTAMFR.R | 234 | 5/13 | 49639/45 | 4.78/4.9 | -1.7±0.13 |
| 53 | N2a12 | Calreticulin precursor  (NP_031617) | K.IDNSQVESGSLEDDWDFLPPKK.I  K.IKDPDAAKPEDWDER.A | 89 | 2/8 | 47965/50 | 4.33/4.4 | -1.6±0.14 |
| 54 | N2a13 | Protein disulfide-isomerase precursor  (NP_035162) | K.VDATEESDLAQQYGVR.G  K.EYTAGREADDIVNWLK.K  K.ILFIFIDSDHTDNQR.I  K.FFPASADR.T  K.FFPASADRTVIDYNGER.T | 249 | 5/12 | 57023/50 | 4.77/4.8 | -1.5±0.14 |
| 55 | N2a14 | Nucleobindin 1, isoform CRA_a  ( NP_001157134) | K.LSQELDFVSHNVR.T  R.DLELLIQTATR.D  R.ELQQAVLQMEQR.K | 97 | 3/7 | 53376/55 | 4.99/5.0 | 1.5±0.12 |
| 56 | N2a15 | Heterogeneousnuclear ribonucleoprotein H  ( NP_067485) | R.STGEAFVQFASQEIAEK.A  R.ATENDIYNFFSPLNPVR.V  R.VHIEIGPDGR.V | 255 | 3/9 | 49168/47 | 5.89/6.3 | 1.5±0.11 |
|  |  |  |  |  |  |  |  |  |

**^a^NCBI accession number of identified proteins is mentioned.**

**^b^ MS/MS data of 3 peptides for each spot was searched against NCBI database in the taxonomy group of *Mus musculus* using Mascot tool.**

**^c^ MP = number of matched peptides with significant ion score; SC = percent sequence coverage. A total of 3 peptides were subjected to MS/MS analysis and the fragment ion data was searched against the data base.**

**^d^ Fold change (±standard deviation) in the expression level after JEV infection.**
