# Supplementary material for: Modulation of Neuronal Proteome Profile in Response to Japanese Encephalitis Virus Infection
Source: PLoS One. 2014 Mar 5;9(3):e90211. doi: 10.1371/journal.pone.0090211 (PMC3943924; doi:10.1371/journal.pone.0090211)
Supplement: Table S2 — Proteins showing differential expression after JEV infection, identified by MS/MS analysis of gel excised spots. (DOC) [file pone.0090211.s008.doc]

**Table S2. List of identified proteins used for protein-protein interaction analysis.**

| **Sr No.** | **Protein** | **Experiment** | **Gene** | **Location*** | **Function** |
| --- | --- | --- | --- | --- | --- |
| 1 | Transitional endoplasmic reticulum ATPase | *In vivo* | Vcp | Cy | Vesicle transport and fusion, 26S proteasome function, assembly of peroxisomes |
| 2 | Alpha fetoprotein | *In vivo* | Afp | Ex | SMAD protein signal transduction, transport |
| 3 | T-complex protein 1 subunit epsilon | *In vivo* | Cct5 | Cy | Chaperonin-mediated protein folding |
| 4 | Aldolase C, fructose-bisphosphate | *In vivo* | Aldoc | Cy | Glycolysis / Gluconeogenesis, putative target of the transcription factor hypoxia-inducible factor (HIF)-1 |
| 5 | 1-Cys peroxiredoxin | *In vivo* | Prdx6 | Cy | Regulation of oxidative stress, Lipopolysaccharide and interferon-gamma induced gene expression |
| 6 | haloacid dehalogenase-like hydrolase domain-containing protein 2 | *In vivo* | hdhd2 | Cy | Hydrolase activity |
| 7 | Lactate Dehydrogenase | *In vivo* | ldhb | Mi | The citric acid (TCA) cycle and respiratory electron transport |
| 8 | F-actin-capping protein subunit beta | *In vivo* | Capzb | Cy | Actin-capping protein is a novel regulator of microtubule stability |
| 9 | pyridoxal phosphate phosphatise | *In vivo* | pdxp | PM | Vitamin B6 metabolism, regulation of cytokinesis |
| 10 | Pyruvate dehydrogenase chain B protein | *In vivo* | Pdhb | Mi | The citric acid (TCA) cycle and respiratory electron transport |
| 11 | ubiquitin carboxyl-terminal hydrolase PGP9.5 | *In vivo* | Uchl1 | Cy | Hydrolase activity, ubiquitin-dependent protein catabolic process |
| 12 | 14-3-3 protein gamma | *In vivo* | Ywhag | Cy | TNF-alpha NF-kB Signaling Pathway, Cell cycle |
| 13 | creatine kinase B-type | *In vivo* | Ckb | Mi | Urea cycle and metabolism of amino group |
| 14 | gamma-actin | *In vivo* | Actg1 | Cy | Focal adhesion, cell communication |
| 15 | gamma-enolase | *In vivo* | Eno2 | Cy | Glycolysis / Gluconeogenesis |
| 16 | alpha-tubulin isotype M-alpha-2 | *In vivo* | Tuba1b | Cy | Gap junction |
| 17 | Calreticulin chain A | *In vivo* | Calr | ER | Antigen processing and presentation, Protein processing in endoplasmic reticulum, Unfolded Protein Response |
| 18 | heat shock protein 70 cognate | *In vivo* | Hspa8 | ER | Response to stress, Protein processing in endoplasmic reticulum |
| 19 | glyceraldehyde-3-phosphate dehydrogenase | *In vivo* | Gapdh | Cy | Glycolysis / Gluconeogenesis |
| 20 | stress-induced-phosphoprotein 1 | *In vivo* | Stip1 | Cy | Axon guidance, L1CAM interactions |
| 21 | proteasome subunit alpha type-1 | *In vivo* | Psma1 | Cy | Antigen processing, Ubiquitination, Proteasome degradation, Apoptosis, Cell Cycle |
| 22 | endoplasmic reticulum protein 29 | *In vivo* | Erp29 | ER | Protein processing in endoplasmic reticulum |
| 23 | Heat shock protein 90, beta (Grp94), member 1 | *In vitro* | Hsp90b1 | ER | Response to stress, Protein processing in endoplasmic reticulum |
| 24 | BiP | *In vitro* | Hspa5 | ER | Response to stress, Protein processing in endoplasmic reticulum |
| 25 | Heat shock protein 8 | *In vitro* | Hspa8 | Cy | Response to stress, Protein processing, Antigen processing and presentation, Golgi Associated Vesicle Biogenesis |
| 26 | peroxiredoxin-4 precursor | *In vitro* | Prdx4 | Mi | Activation of the transcription factor NF-kappaB, Selenium Pathway |
| 27 | isocitrate dehydrogenase [NAD] subunit alpha | *In vitro* | idh3a | Mi | The citric acid (TCA) cycle and respiratory electron transport |
| 28 | gamma-actin | *In vitro* | Actg1 | Cy | Focal adhesion, cell communication |
| 29 | nucleophosmin isoform 1 | *In vitro* | Npm1 | Cy | Aurora B signaling, Cell Cycle |
| 30 | Protein disulfide isomerase associated 6 | *In vitro* | Pdia6 | ER | Unfolded Protein Response, Protein processing in endoplasmic reticulum |
| 31 | Vimentin | *In vitro* | Vim | Cy | Maintains cell shape, integrity of the cytoplasm, stabilizing cytoskeletal interactions |
| 32 | Atp5b protein | *In vitro* | Atp5b | Mi | ATP synthesis, Oxidative phosphorylation |
| 33 | calreticulin precursor | *In vitro* | Calr | ER | Antigen processing and presentation, Protein processing in endoplasmic reticulum, Unfolded Protein Response |
| 34 | nucleobindin 1, isoform CRA_a | *In vitro* | Nucb1 | Go | Golgi calcium homeostasis, Ca(2+)-regulated signal transduction events |
| 35 | heterogeneous nuclear ribonucleoprotein H | *In vitro* | Hnrnph2 | Nu | pre-mRNA processing, mRNA metabolism and transport |

*Cy-Cytoplasm, Ex- Extracellular, Mi-Mitochondria, PM- Plasma membrane, ER- Endoplasmic reticulum, Go-Golgi, Nu- nucleus.
