# Supplementary material for: Modulation of Neuronal Proteome Profile in Response to Japanese Encephalitis Virus Infection
Source: PLoS One. 2014 Mar 5;9(3):e90211. doi: 10.1371/journal.pone.0090211 (PMC3943924; doi:10.1371/journal.pone.0090211)
Supplement: Table S3 — List of identified proteins used for protein-protein interaction analysis. (DOC) [file pone.0090211.s009.doc]

| **Sr. No.** | **Proteins** | **Molecular weight** | **Dilution for Western Blot** |
| --- | --- | --- | --- |
|  | Transitional endoplasmic reticulum ATPase (VCP) | 97 kda | 1:2000 |
|  | T-complex protein 1 subunit epsilon | 60kDa | 1:10000 |
|  | Aldolase C, fructose-bisphosphate | 44kDa | 1:1000 |
|  | 1-Cys peroxiredoxin | 25kDa | 1:2000 |
|  | L-lactate dehydrogenase B chain | 37kDa | 1:10000 |
|  | Pyridoxal phosphate phosphatase | 32kDa | 1:200 |
|  | Ubiquitin carboxyl-terminal hydrolase PGP9.5 | 26.8 kDa | 1:1000 |
|  | Rho GDP dissociation inhibitor (GDI) alpha | 23kDa | 1:2000 |
|  | Creatine kinase B-type | 43kDa | 1:10000 |
|  | Gamma-enolase | 47kDa | 1:1000 |
|  | Calreticulin chain A | 63kDa | 1:1000 |
|  | Proteasome subunit alpha type-1 | 29kDa | 1:1000 |
|  | BiP | 75kDa | 1:1000 |
|  | Peroxiredoxin-4 precursor | 29kDa | 1:2000 |
|  | Nucleophosmin isoform 1 | 33kDa | 1:1000 |
|  | Protein disulfide isomerase associated 6 | 57kDa | 1:1000 |
|  | Atp5b protein | 57kDa | 1:1000 |
|  | Heat shock protein 60 | 60kda | 1:20000 |
|  | Nucleobindin 1, isoform CRA_a | 54kDa | 1:500 |
|  | Heterogeneous nuclear ribonucleoprotein H | 53kDa | 1:10000 |
|  | Endoplasmic reticulum protein 29, isoform CRA_a | 29kDa | 1:2500 |
|  | Heat shock protein 90, beta (Grp94), member 1 | 90kDa | 1:10000 |
|  | Pyruvate dehydrogenase chain B protein | 39kDa | 1:1000 |
|  | Ubiquitin carboxyl-terminal hydrolase isozyme L3 | 26.9 kDa | 1:1000 |
|  | 14-3-3 protein gamma | 28kDa | 1:1000 |
|  | Tubulin beta-2B chain | 53kDa | 1:1000 |
|  | Heat shock protein 70 cognate | 70kDa | 1:1000 |
|  | Stress-induced-phosphoprotein 1 | 63kDa | 1:10000 |
